# The representation of child–parent relation: validation of the Italian version of the child–parent relationship scale (CPRS-I)

**DOI:** 10.3389/fpsyg.2023.1194644

**Published:** 2023-09-20

**Authors:** Teresa Rinaldi, Ilaria Castelli, Nicola Palena, Andrea Greco, Robert Pianta, Antonella Marchetti, Annalisa Valle

**Affiliations:** ^1^Department of Psychology, Università eCampus, Novedrate, Italy; ^2^Department of Humanities and Social Sciences, Università degli Studi di Bergamo, Bergamo, Italy; ^3^Center for Advanced Study of Teaching and Learning, Curry School of Education and Human Development, University of Virginia, Charlottesville, VA, United States; ^4^Research Unit on Theory of Mind, Department of Psychology, Università Cattolica del Sacro Cuore, Milan, Italy

**Keywords:** child–parent relation, attachment, parent representation, Italian validation, conflict, closeness, dependency

## Abstract

This study proposes a psychometric validation of the Italian version of the Child–Parent Relationship Scale (CPRS) developed by Pianta in 1992. Based on attachment theory, the scale assesses parents’ relationship perceptions with their own child and comprises three scales: Closeness, Conflict, and Dependency. A sample of 501 parents (188 fathers and 313 mothers) completed 30 items of the Italian version of the Child–Parent Relationship Scale (CPRS-I) online, but only 437 answered 85% of the entire protocol; hence, the analyses only focused on 437 participants. The first analysis of the original theoretical model revealed poor fit, item loadings, and internal consistency. Therefore, a follow-up analysis was conducted. Exploratory and confirmatory analyses with a split sample (EFA = 218; CFA = 219) confirmed the original three-factor structure of the Italian sample, although some items were eliminated. The validity and reliability of the Italian version of the CPRS-I were also verified by correlating the above three factors with measures of adult attachment styles and children’s internalizing and externalizing behaviors. The CPRS-I showed significant correlations with all tested constructs, in line with those found by Driscoll and Pianta for the short form of the scale. Our results confirm that the CPRS-I has the same structure as the original scale; therefore, it can be a useful tool for assessing parents’ perceptions of their relationship with their children. The implications for educational and clinical settings are also discussed.

## 1. Introduction

The characteristics of the affective relationships between children and their family caregivers play a central role in their development in terms of socio-emotional skills, mental health, language and cognitive skill, mentalization abilities (Repetti et al., 2002; Tamis-LeMonda et al., 2004; Fonagy et al., 2016), positive relationships with peers (Solak Arabaci and Demircioğlu, 2021), academic achievement, and school adjustment (Pianta and Steinberg, 1992; Pianta and Stuhlman, 2004; Pianta, 2019). The main framework used to analyze affective relationships is the attachment theory (Bowlby, 1969), according to which the closeness/exploration behavioral dynamic is the first relevant experience of caregiver availability and sensitivity. Based on this experience, infants adapt their relational behavior to their caregiver responses. During development, they internalize these reciprocal behaviors and build internal working models—representations of the attachment bonds that guide the individual in constructing new affective bonds with other significant partners across their life-span, such as extra-familiar educators, teachers, romantic partners.

In the parent–child relationship, parents’ behaviors are guided by an underlying caregiving behavioral system (Bowlby, 1982), including a broad array of behaviors with two main functions: providing a safe haven to support the attachment behavior of the child and providing a secure base for the child to support her/his exploration (Feeney and Woodhouse, 2016). According to Driscoll and Pianta (2011), parents’ internal representations of the relationship are components of this caregiving system and contributes to shaping the quality of the relationship with the child (Chow et al., 2017); for example, in a close relationship, the parent functions as a safe haven, whereas in a dependent relationship, the parent does not promote the exploration and autonomy of the child. In this framework, Dyer et al. (2017) argued that parental representations of the relationship with the child are well-described as closeness and conflict. Driscoll and Pianta (2011) defined closeness as warmth, affection, and open communication of emotions and considered it an important predictive factor of a child’s social competence and adjustment from early childhood to adolescence. Conflict is defined as behavioral opposition or overt disagreement, usually present in the parent–child bond (Maccoby, 1992). In younger children, a high level of conflict refers to discordant interactions and a lack of security in the relationship between adults and children. In adolescence and adulthood, conflict management differs according to attachment style: in secure attachment, conflict management is characterized by positive negotiation to reach a compromise; in insecure attachment, individuals engage in whining, nagging, hostile, and aggressive behavior (La Valley and Guerrero, 2012). According to Pianta’s perspective (Pianta, 1992; Koomen et al., 2012), dependency also contributes to describe the affective relationships. Dependency refers to attachment behavior, such as seeking contact and attention from adult caregivers to elicit caregiving responses. A high level of dependency entails overreliance on the parent, excessive and inappropriate help-seeking, and clinging behavior toward that parent (Verschueren and Koomen, 2012); thus, limiting exploration of the world and the possibility of building social interactions with peers.

### 1.1. Parent–child quality assessment

The quality of the parent–child relationship can be studied by focusing on each of its many components, such as parents’ sensitivity, emotional availability, and stress (Foran et al., 2020), or by focusing on the characteristics of the relationship itself, as proposed by Driscoll and Pianta (2011) with the Child–Parent Relationship Scale (CPRS). Regarding the latter category, the literature proposes self-report questionnaires and scales for parents perspective, such as the Parent–Child Interaction Questionnaire Revised (Lange et al., 2002), composed of Conflict resolution and Acceptance factors; the Parent–Child Relationship Inventory (Gerard, 1994), assessing parental attitudes toward parenting, parenting behaviors, and children; the Parent–Child Relationship Questionnaire (Furman and Giberson, 1995), assessing warmth, closeness, disciplinary warmth, power assertion, and possessiveness; the Parent-Adolescent Relationship Scale (Burke et al., 2021), composed of connectedness, shared activities, and hostility factors. Although some of these tools include factors close to or overlapping those of the CPRS (e.g., closure/connectedness and conflict/hostility), none have focused on the three CPRS factors. In addition, a search for closeness, conflict, and dependency, separately, does not reveal many assessment tools. Recently, to assess closeness, Chung et al. (2022) created three items based on the Driscoll and Pianta perspective, and Fang et al. (2021) proposed three questions on emotional and behavioral connectedness. Regarding the relational conflict, near to the tools assessing violent and nonviolent forms of conflict between parent and child, such as the Parent–Child Conflict Tactics Scale (Straus et al., 1998), the literature proposes the Parent–Child Conflict Scale of the Parental Environment Questionnaire (Elkins et al., 1997; Wong et al., 2023), assessing how each family member perceives the level of conflict in her/his relationship with the other family member, or the Conflict Resolution Styles Questionnaire (Peterson, 1990; Feeney, 2006) assessing avoidance, attack, and problem-solving in the conflict.

The tools reported here highlight the complexity of assessing the quality of parent–child relationships, which can be described from multiple perspectives. In our opinion, the CPRS allows us to focus simultaneously on three important facets of the relationship indicated by the Pianta’s theory, making it possible to describe different aspects of the parent’s perception of the relationship with a single tool.

### 1.2. The child–parent relationship scale (CPRS)

The CPRS (Pianta, 1992) is a self-report scale assessing parents’ perceptions of their relationship with their child and is considered a key indicator of the quality of the parent–child relationship. The scale measures both positive and negative aspects of the parent–child relationship through the closeness, conflict, and dependency dimensions. This CPRS structure was derived from the Student–Teacher Relationship Scale (STRS; Pianta, 2001), which assesses the perception of the student–teacher relationship along the same three dimensions. From a multiple-caregiver perspective, the relationship with the parent and with the teacher are certainly different, but they show some similarities that allow both to be considered attachment relationships. These relationships are different for exclusivity, duration, emotional investment, and type of caregiving behavior. However, their similarity results from the caregiver acting as a safe haven and a secure base in both cases. Moreover, the pattern of separation–reunion behavior is similar, and harmony, comfort-seeking, resistance, and avoidance are dimensional characteristics in both relationships (Verschueren and Koomen, 2012). Additionally, the student–teacher relationship can be considered an attachment bond (Valle et al., 2019, 2022), temporary “used” by the children when the parent is unavailable.

Regarding the factors assessed by the CPRS, Closeness is considered a positive aspect of the relationship and is evaluated through items, such as “If upset, my child will seek comfort from me” (Item 3) and “My child spontaneously shares information about himself/herself” (Item 10). Conflict is considered a negative aspect of the relationship, and is assessed with items, such as “My child and I always seem to be struggling with each other” (Item 2) and “Dealing with my child drains my energy” (Item 21). Finally, dependency is considered a stressful feature of the attachment relationship, and is assessed with items, such as “My child reacts strongly to separation from me” (Item 9) and “My child is overly dependent on me” (Item 11).

In the original version devised by Pianta (1992), the CPRS-long form (CPRS-LF) is composed of 30 items, each describing a specific behavior that the child shows with the parent. The adult indicates her/his responses on a five-point Likert scale, with answer options ranging from “Definitely does not apply” to “Definitely applies.”

Two validation studies of the CPRS-LF were proposed for its Turkish translation with two different samples comprising mothers and fathers. The first study involving mothers was conducted by Akgun and Yesilyaprak (2010). Using principal component analysis, the authors individuated two factors, Conflict (14 items) and Positive Relationship (10 items), with alpha values of 0.85 and 0.73 for each factor, respectively. The conflict factor consisted of 12 items belonging to the original conflict dimension plus two items belonging to the original dependence factor. The second study by Uzun and Baran (2019) investigated the internal consistency and stability of the CPRS-LF among fathers. Through an exploratory factor analysis, they derived a scale composed of 23 items organized into three factors: Positive Relationships (10 items), Incompatibility (7 items), and Conflict (6 items). Cronbach’s alpha reliability coefficients were 0.76 for the positive relationships, 0.61 for incompatibility, 0.62 for conflict factors, and 0.71 for the whole instrument. This structure explained 36.8% of the total observed variance. As suggested by Escalante-Barrios et al. (2020), the two aforementioned studies by Akgun and Yesilyaprak (2010) and Uzun and Baran (2019) showed that the factorial structure of the CPRS-LF differed between the United States and Turkish cultures, as well as between mothers and fathers.

Adapting this type of assessment to different cultures was pursued more systematically using the short form of the CPRS (CPRS-SF) developed by Driscoll and Pianta (2011). The CPRS-SF comprises 15 items: seven in the closeness factor and eight in the conflict factor. Both mothers and fathers completed the CPRS-SF when their children were 54 months old and in first grade (between 6 and 7 years). The results showed that maternal and paternal ratings of both closeness and conflict were stable during the period considered and that mothers showed higher levels of closeness and conflict than fathers in both surveys. More recently, Dyer et al. (2017) confirmed the two-factor structure of the scale in a sample of non-resident fathers, showing the validity of the CPRS-SF in the US context. Simkiss et al. (2013) validated the CPRS-SF in a UK sample, confirming the two-factor structure and eliminating one item (item 4), assessing the perception of avoidance of physical contact and affection. The same result was achieved by Escalante-Barrios et al. (2020) in the Turkish version of the scale with low-income parents.

One of the most important differences between the CPRS-LF and the CPRS-SF seems to be the dependence factor, which is not included in the SF because of its low reliability (Zhang and Chen, 2010). Dependency is classically considered a relatively stable, individual trait (see Ainsworth, 1969) that is able to generate stress in the adult, thus impacting her/his caregiving behaviors, but not necessarily related to attachment security (Howard, 2010). More recently, Verschueren and Koomen (2021) proposed considering dependency as a relational construct that varies in quality across different caregiving relationships (i.e., the relationship with the mother, father, and teacher) and caregiver behaviors.

In line with this last proposal, we decided to validate the LF of the CPRS in the Italian context because we consider the dependency level showed by the child and perceived by the parent as a result of the specific caregiver–child relationship.

### 1.3. Parent–child quality relationship from a cultural perspective

The validation of the CPRS in Italy is consistent with the increasing interest in parent–child relationships from a cultural perspective. In the attachment framework, a large part of the literature argues for the existence of fundamental principles in cultures: all children look for an adult figure to attach themselves to (the universality hypothesis), and the adult has to respond to infant signals in order to promote safety, sense of security, and emotional support to children (the sensitivity hypothesis), thus promoting their social–emotional development (the competence hypothesis; Mesman et al., 2016). Nevertheless, some literature suggests not only that the parent–child relationship can be influenced by cultural factors, such as caregiving practices and social expectations, but that the above-cited principles are not universal because they depend on the means that specific cultures attribute to this relationship. Starting with the difference between studies in Western middle-class people and non-Western traditional people, Keller (2018) analyzed one of the most important principles of security attachment—the caregiver’s sensitivity and responsiveness. In Western cultures, sensitivity is demonstrated through verbal input (taken with the child), whereas, in Eastern cultures, mothers usually prefer physical proximity. The author supposes that this difference reflects a deeper difference in caregiving behaviors, parenting representations, and beliefs. In Western cultures, the baby is considered an independent intentional agent who develops autonomy mainly in dyadic relationships within the family, whereas, in other cultures, families socialize with infants to follow the directives of caregivers in multiple caregiving contexts where different partners attend to different attachment functions. Keller (2018) denied the universality of attachment, considering care practices and the culturally determined parent–child relationship. This hypothesis underlined Trommsdorff (2006) studies—according to the author, in Western individualistic countries, development is characterized by ever-greater autonomy, whereas in Eastern collectivistic countries, development is considered the capacity to fulfill familiar roles and responsibilities.

Additionally, in the Western cultures—also considered individualistic cultures—a difference in the families is demonstrated; in Mediterranean countries, the families are named “strong-families,” characterized by closer and more intense relationships and emotional bonds than the “weak families” in the US and northern Europe (Giannotta et al., 2013). This can explain the fact that dependency on the family is perceived differently in Italy than in Anglo-Saxon countries; in fact, dependency on parents is considered the normal condition of Italian children, such that autonomy from the original family is reached very late with respect to Anglo-Saxon or northern European countries (Mancinelli et al., 2021). This is also evident in parenting; Italian mothers are more intrusive, have less autonomy with respect to English mothers, display a high level of control and protection, and show more warmth than English mothers (Raudino et al., 2013)—highlighting all cues of a dependent relationship. Despite these specificities, Western cultures aim to promote children’s independence and autonomy as they grow up, unlike Eastern cultures, in which interdependence and bonds with adults and peers are promoted. In view of these remarks, we are interested in the role of dependency in Italian child–parent relationships, assuming that it emerges in the CPRS-I, as theorized for its original version.

Another culturally related question is the father’s role in parent–child caregiving. In Western countries, fathers have become increasingly involved in the care of children from infancy over the past few decades; therefore, they are considered attachment figures in their own right. In addition to the functions of a safe haven and secure base, the father plays a role in the dynamic (Grossmann et al., 2002) characterized by the capacity to excite and destabilize the child during play while providing safety and security. This dynamic indicates that behaviors related to fathers’ sensitivity are different from those of mothers, but research concludes that they are equally important in the construction of an attachment relationship (Cabrera, 2020; Van Bakel and Hall, 2020). This sensitivity involves a distinctive level of closeness and dependency. In a secure attachment, the father is neither too close nor too far from the child, so he can control the child during her/his autonomous play and protect her/him in case of danger. In addition, conflict is considered a fundamental characteristic of the father–child relationship. Referring to the identity theory, Dyer et al. (2017) affirmed that the perceived conflict level reflects a dissonance between the relationship characteristics and the father’s expectations, that is, the performance standard that is in part culturally defined. Thus, the level of perceived conflict can be a cue for the father’s sense of adequacy in his parental role.

### 1.4. Aims and hypotheses

This study aims to test the psychometric validity and reliability of the Italian version of the CPRS-LF (CPRS-I) using a cohort of Italian parents. Specifically, we aim to:

1. Test factorial validity (using confirmatory factor analysis (CFA) of the CPRS-LF). We hypothesize that the CPRS-I would replicate the three-factor structure of the original scale. Although the factorial structure of the Turkish validations of the CPRS-LF is different from the original, we expect the dimensions individuated by the CPRS-I to be the same as those of the original scale. Turkey seems to have characteristics of both individualistic and collectivistic cultures (Escalante-Barrios et al., 2020), whereas Italy is considered an individualistic culture (Mancinelli et al., 2021) similar to the US, thus assuming the same scale structure.
2. Explore the measurement invariance of the CPRS-I regarding parent’s and daughters’/sons’ sex by employing multigroup confirmatory factor analysis.
3. Test the assessment’s reliability (through internal consistency) and concurrent and convergent validity (through Pearson’s correlation) by examining associations between the CPRS-I and parents’ attachment style (assessed by the Attachment Style Questionnaire, ASQ) and daughters’/sons’ behavioral problems (assessed by the Child Behavioral Check List, CBCL). In light of the link between attachment and parent–child relationship quality and caregiving and between attachment style and family functioning (López-de-la-Nieta et al., 2021), we hypothesize a correlation between the CPRS-I and the parent’s attachment style; more specifically, we hypothesize positive correlations between conflict and dependency, and insecure attachment styles (discomfort with closeness, need for approval, preoccupation with relationships and relationships as secondary ASQ dimensions) and a negative correlation between closeness and the discomfort with closeness ASQ dimensions.

Moreover, we hypothesize that closeness negatively correlates with children’s behavioral problems, and that conflict and dependency are positively correlated with children’s behavioral problems, as found by Driscoll and Pianta (2011).

## 2. Materials and methods

### 2.1. Participants

In total, 505 Italian parents of school-aged children and adolescents (6–18 years) participated in the study: 188 (37%) were fathers, and 313 (62%) were mothers (4 answers are missed). The age range of the patients was 32–74 years. A total of 485 participants (96.04%) were biological parents of their children, and 13 (2.57%) were adoptive parents. Moreover, 261 (51.68%) participating parents declared having a son, and 242 (47.92%) declared having a daughter. 68 (13.46%) parents did not complete the questionnaire and were excluded from the analysis. The final sample consisted of 437 participants.

The characteristics of the participants are shown in the table below (Table 1).

**Table 1:** Demographic information.

		Number (percentage)
Employment status	Employment status	445 (88.12%)
	Unemployed	17 (3.36%)
	Housewives	29 (5.74%)
	Retired	10 (1.98%)
Marital status	Married	413 (81.78%)
	Single	42 (8.31%)
	Divorced	45 (8.91%)
	Widowed	1 (0.19%)
Cohabitation	Live with the spouse and child/children	453 (89.70%)
	Live with the child/children	37 (7.32%)
	Live alone	2 (0.39%)
	Live only with the partner	2 (0.39%)
	Live with relatives other than partner and child/children	2 (0.39%)
Educational level	Education lower than a high school diploma	271 (53.66%)
	High-school diploma	145 (28.71%)
	University degree	39 (7.72%)
	Post-graduate	271 (53.66%)

### 2.2. Measures

#### 2.2.1. Sociodemographic information

All participants were asked to provide sociodemographic information, such as sex, year of birth, education level, marital status, employment status, and residence type. The inclusion criteria were legal age (i.e., over 18 years in Italy) and having at least one child between the ages of 6 and 18 years.

#### 2.2.2. Adult attachment style

The adult attachment style was assessed by the ASQ (Feeney et al., 1994) in the Italian version of Fossati et al. (2003, 2007). The ASQ is a 40-item self-administered questionnaire designed to measure the five dimensions of adult attachment on a 6-point scale, ranging from 1 (totally disagree) to 6 (totally agree). The five dimensions of attachment with the corresponding attachment styles (as indicated by López-de-la-Nieta et al., 2021) included: Confidence (8 items; range 8–48; α = 0.69), corresponding to the secure attachment; Discomfort with Closeness (10 items; range 10–60; α = 0.68), corresponding to the avoidant style; Need for Approval (7 items; range 7–42; α = 0.69), corresponding to the preoccupied style; Preoccupation with Relationships (8 items; range 8–48; α = 0.64), corresponding to the anxious/ambivalent and preoccupied style; Relationships as Secondary (7 items; range 7–42; α = 0.73), corresponding to the dismissing style.

#### 2.2.3. Children’s behavioral and emotional problems

Parents’ perceptions of their children’s emotional and behavioral problems in children aged 6–18 years were assessed using the Child Behavior Checklist (CBCL/6–18; Achenbach and Rescorla, 2001) in the Italian translation of Frigerio (2001). The CBCL 6–18 is a 113-item parent report measure designed to detect internalizing and externalizing problems among children and adolescents. It can be completed in person or online by the parents on a 3-point Likert scale (0 = “Absent,” 1 = “Occurs sometimes,” 2 = “Occurs often”). The score was assessed by assigning one point to each answer. The CBCL comprises eight subscales: anxiety/depression, depression, somatic complaints, social problems, thought problems, attention problems, rule-breaking behavior, and aggressive behavior. These subscales can be grouped into two higher-order factors: internalization and externalization. Scoring was obtained by summing up all the problem items from a minimum of 0 to a maximum of 226. Internalizing behaviors were calculated by summing up the anxious/depressed, depressed, and somatic complaints subscales (*α* = 0.90), while externalizing behaviors were calculated by summing up the rule-breaking behavior and aggressive behavior subscales (*α* = 0.94).

### 2.3. Procedure

Data were collected through an online survey hosted on the Qualtrics platform between March 2019 and January 2020.

The participants were administered using a protocol composed of the Italian version of the original English CPRS,^1^ followed by the above-mentioned measures translated from English to Italian—by a professional translator and a psychologist with a back-translation procedure—to ensure that the meaning of each sentence or item was accurately reflected. Once the study protocol was implemented and completed, a survey link was presented to university courses at the Department of Human and Social Sciences at the University of Bergamo and the Faculty of Education at the Catholic University of the Sacred Heart of Milan. The same link was sent to the authors’ personal contacts and other participants using a snowball sampling method. In addition to providing a survey link, the participants were presented with all the necessary information, including the study purpose, instructions, and survey duration, which was estimated in approximately 30 min. On the first page of the survey, participants were informed about personal data processing, and only those who provided informed consent were included in the data collection. All participants were treated in accordance with the ethical guidelines for research provided by the Declaration of Helsinki, American Psychological Association, and Italian Psychological Association. According to APA ethical standards, this study was approved by the local ethics committee of the Department of Psychology of the Catholic University of the Sacred Heart of Milan. Participants provided sociodemographic information first and then completed the CPRS-I, ASQ, and CBCL, in the same order.

### 2.4. Statistical analyses

First, we explored the normality of the data according to West et al. (1995), who suggested considering items whose skewness and kurtosis did not exceed |2| and |7|, respectively, as normal.

We then focused on the factorial structure of the CPRS. However, when a scale is translated into a different language and applied to a cultural context different from the original version, there may be differences in its latent structure. Therefore, we first conducted a CFA on the original model to test its fit. However, we also assessed the latent structure through exploratory analysis, followed by confirmatory analysis. We first randomly divided the sample into two subsamples. One subsample (Subsample A) was used to conduct parallel analysis and subsequent Exploratory Factor Analysis (EFA; *n* = 218). In order to aid in deciding how many factors should be retained in the study we considered the following: (1) the subjectivity of deciding how many factors to retain through exploring the screen plot, and (2) the decision rule, “eigenvalue is sgreater than one,” is associated with the number of items (Greco et al., 2022). Thus, made use of Horn’s method to conduct a Parallel Analysis (Horn, 1965). We then conducted an EFA, in which the Kaiser-Meyer-Olkin (KMO; which should be at least 0.50) and Bartlett’s test of sphericity (which should be significant) were run to ensure that the data were suitable for the analyses (Horn, 1965). We employed the principal axis factor and promax oblique rotation because theoretical reasons indicate that the CPRS factors are related. Initially, all 30 CPRS items were included in the EFA. Items showing loadings <0.32, items showing loadings >0.32 on more than one factor, and items whose secondary loading was higher than half the primary loading were eliminated in a stepwise fashion (Tabachnick and Fidell, 2001), whereas those that did not were retained. Furthermore, we focused on communality to verify the item’s quality (items with communality higher than 0.25 were retained).

Once a satisfactory factor structure was reached, CFA was conducted on the second subsample (subsample B, *n* = 219). We adopted a Maximum Likelihood estimator and relied on the following indices to test the fit of the CFA models: chi-square test statistics, root-mean-square error of approximation (RMSEA), and standardized root-mean-square residual (SRMR). Further, in cases where the RMSEA of the null model was >0.158, we also reported the Comparative Fit Index (CFI) and Tucker-Lewis Index (TLI) as null RMSEA <0.158 makes the CFI and TLI non-interpretable (Kenny et al., 2015). RMSEA and SRMR ≤0.08, CFI and TLI ≥0.90 and non-significant *χ^2^* were interpreted as a reasonable fit.

Multigroup CFA was conducted on the entire dataset to test for sex invariance (both parents and children). Three different models were obtained and compared: (i) configural invariance, where the factor structure was assumed to be the same across groups; (ii) metric invariance, where loadings were also assumed to be the same across groups; and (iii) scalar invariance, where, in addition to the previous intercepts, were also assumed to be the same. We concluded that the tool was sex invariant when the changes in RMSEA were ≤ 0.015, ≤ 0.030 for SRMR, and for those cases where we also reported the CFI and TLI, their changes were ≤ 0.010 (Cheung and Rensvold, 2002; Chen, 2007).

In addition, we explored the reliability and validity of the entire dataset. Cronbach’s α and McDonald’s α.60 were deemed indicative of acceptable internal consistency (Nunnally and Bernstein, 1994). Validity was tested using Pearson’s correlations between CPRS scores and both CBCL and ASQ scores. Finally, we explored the effect of sex (both parents and children) on CPRS scores using a MANOVA.

Parallel, correlation, and internal consistency analyses were conducted using Jamovi version 1.6 (The Jamovi Project, Sydney, Australia). Descriptive statistics, EFA, and MANOVA were performed using SPSS version 27 (IBM Corp., Armonk, N.Y., United States). Confirmatory Factor Analysis (CFA) and Measurement Invariance were conducted in R version 4.0.3 (R Core Team, 2020) and R studio (version 1.3.1093; RStudio Team, 2020 using the R Package Lavaan; Rosseel, 2012).

## 3. Results

### 3.1. Preliminary analyses

The average scores of responses to the CPRS items ranged from 1.86 to 4.70 (Sdmin = 1.10; Sdmax = 1.94). None of the items was distributed non-normally (Skewness_min_ = 0.015, Skewness_max_ = 1.97; Kurtosis_min_ = 0.002, Kurtosis_max_ = 3.22).

### 3.2. Confirmatory factor analysis of the original model

CFA of the entire dataset, assuming the original model, showed a satisfactory fit, *χ^2^*(296) = 723.26, *p* < 0.001, RMSEA = 0.057, SRMR = 0.068. The CFI and TLI were not reported as null models (RMSEA < 0.158). However, there were items with non-significant loadings, several had low loadings, and only the factor “Conflict” showed satisfactory reliability (Table 2). Therefore, we tested a revised version of CPRS.

**Table 2:** Items significance and loadings of the original CPRS model.

Item	*p*	Loadings
Closeness (α = 0.60, ω =0.62)		
CPRS_29	–	0.71
CPRS_01	0.15	0.08
CPRS_03	<0.01	0.43
CPRS_05	0.96	0.00
CPRS_08	<0.01	0.20
CPRS_10	<0.01	0.64
CPRS_13	<0.01	0.17
CPRS_16	<0.01	0.52
CPRS_22	0.12	0.09
CPRS_30	<0.01	0.48
Conflict (α = 0.82, ω =0.82)		
CPRS_02	<0.01	0.53
CPRS_12	<0.01	0.58
CPRS_14	<0.01	0.59
CPRS_17	<0.01	0.50
CPRS_18	<0.01	0.38
CPRS_19	<0.01	0.42
CPRS_21	<0.01	0.58
CPRS_23	<0.01	0.49
CPRS_24	<0.01	0.56
CPRS_25	<0.01	0.62
CPRS_27	<0.01	0.44
CPRS_28	<0.01	0.60
Dependency (α = 0.44, ω =0.50)		
CPRS_06	–	0.29
CPRS_09	<0.01	0.68
CPRS_11	<0.01	0.57
CPRS_26	<0.01	0.26

### 3.3. Factor structure of the revised CPRS scale

**Figure 1:**
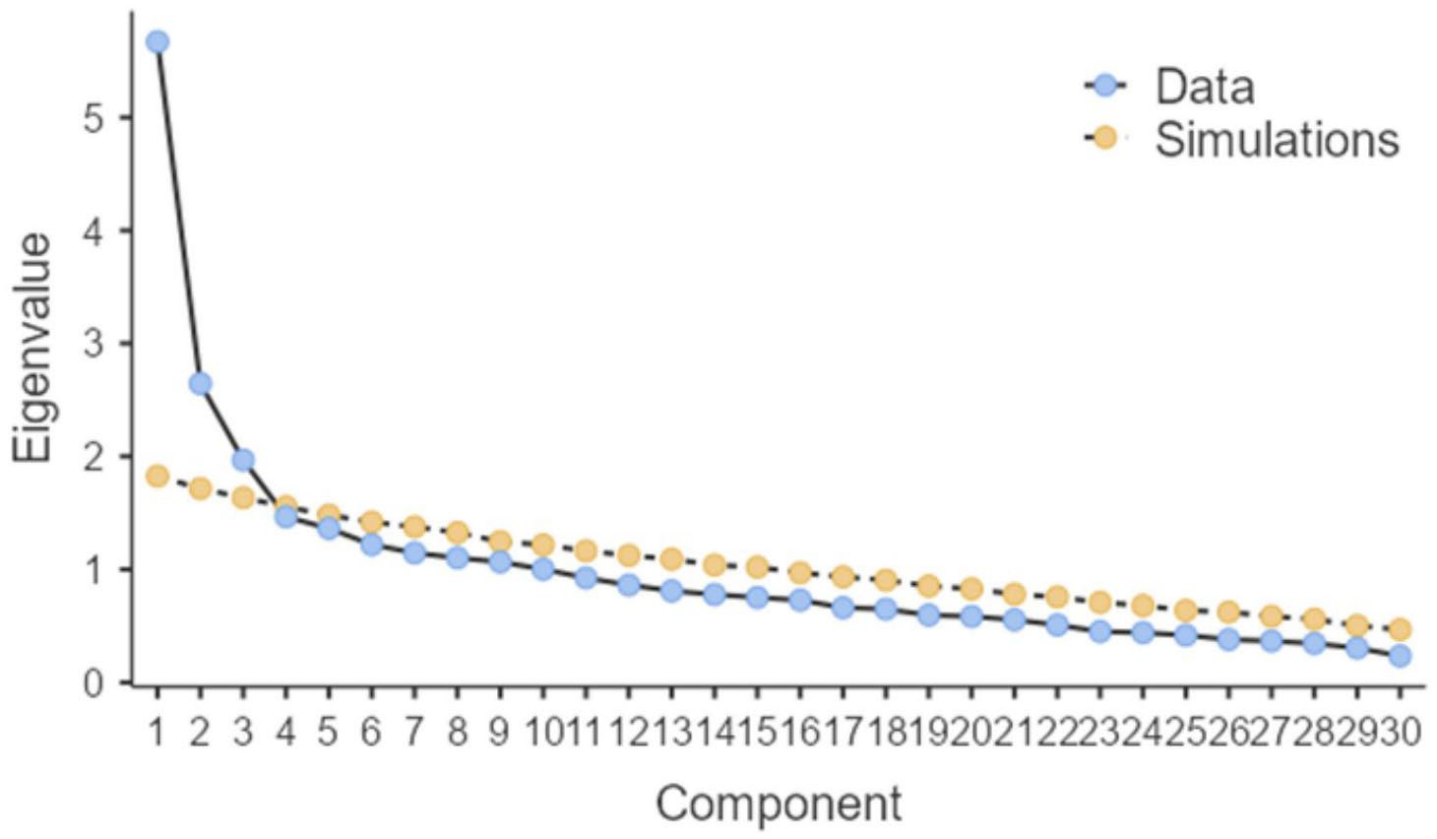
Screen plot of the parallel analysis.

Data from Subsample A—including all 30 items–were used to perform Parallel Analysis and an EFA. Parallel Analysis suggested a three-factor solution (Figure 1). Concerning EFA, Bartlett’s test of sphericity, *χ^2^*(435) = 1657.43, *p* < 0.001, and KMO, 0.78, indicated that the data were suitable for EFA. Based on the results of the Parallel Analyses, we conducted an EFA, forcing a three-factor solution. The initial pool of 30 items was reduced to 23 items after subsequent factor analyses were conducted in a stepwise manner. Two items were excluded because they showed low loadings, cross-loadings, and low communality (CPRS_01: “I share an affectionate, warm relationship with my child”; CPRS_22: “I’ve noticed my child copying my behavior or ways of doing things”). Two items were excluded because they showed cross-loadings (CPRS_08: “When I praise my child, he/she beams with pride”; CPRS_27: “My child whines or cries when he/she wants something from me”). One was excluded because it showed cross-loading and low communality (CPRS_05: “My child values his/her relationship with me”), one because showed low loading and communality (CPRS_13: “My child tries to please me”), and one because showed low communality (CPRS_26: “I often think about my child when at work”). The factor loadings of the three-factor exploratory measurement model for the CPRS items are presented in Table 2. The first factor explained 17.32% of the variance and included 14 items measuring conflict between parents and children. The second factor explained 7.69% of the variance and included five items measuring closeness between parents and children. The last factor explained 6.71% of the variance and included four items measuring dependence. Hence, the model explained 31.72% of the variance. As reported in Table 2, none of the items showed loadings <0.32.

A CFA was conducted on subsample B based on the EFA loadings and showed a good fit, *χ^2^*(227) = 433.15, *p* < 0.001, RMSEA = 0.064, SRMR = 0.069. The CFI and TLI were not reported as null models RMSEA < 0.158. The item loadings for the CFA are reported in Table 3.

**Table 3:** Factor loadings from the EFA and CFA.

	Subsample A – EFA			Subsample B – CFA
	Conflict	Closeness	Dependency	
Loadings				
CPRS_14	**0.67**	0.09	−0.03	0.59^*^
CPRS_12	**0.65**	0.07	0.08	0.57^*^
CPRS_02	**0.61**	0.04	−0.11	0.56^*^
CPRS_23	**0.58**	0.05	0.02	0.40^*^
CPRS_25	**0.57**	−0.24	0.00	0.59^*^
CPRS_24	**0.54**	−0.15	0.03	0.50^*^
CPRS_20	**0.52**	0.19	−0.07	0.34^*^
CPRS_21	**0.51**	0.00	0.02	0.62^*^
CPRS_07	**0.49**	−0.08	−0.02	0.52^*^
CPRS_28	**0.47**	−0.23	0.13	0.49^*^
CPRS_17	**0.46**	−0.01	0.04	0.50^*^
CPRS_19	**0.45**	0.03	−0.01	0.46^*^
CPRS_04	**0.45**	0.01	−0.07	0.55^*^
CPRS_06	**0.34**	0.11	0.15	0.37^*^
CPRS_10	0.20	**0.70**	−0.09	0.64^*^
CPRS_29	−0.01	**0.66**	−0.02	0.71^*^
CPRS_03	0.06	**0.51**	0.14	0.42^*^
CPRS_30	0.00	**0.49**	−0.03	0.47^*^
CPRS_16	−0.09	**0.40**	0.12	0.61^*^
CPRS_11	−0.10	0.01	**0.69**	0.55^*^
CPRS_09	−0.01	0.11	**0.65**	0.58^*^
CPRS_18	0.13	0.02	**0.52**	0.47^*^
CPRS_15	−0.03	−0.08	**0.51**	0.40^*^

^*^p < 0.001. Bold items indicate factor membership.

### 3.4. Reliability, measurement invariance, validity, and relationship with outcome variables

The following analyses were conducted on the entire sample. Internal consistency was good for conflict (*α* = 0.83, ω = 0.84) and closeness (*α* = 0.68, ω = 0.69), and acceptable for dependency (*α* = 0.62, ω = 0.63). Multigroup CFA showed that the model had scalar invariance for both parents’ and children’s sex. Indeed, changes in RMSEA never exceeded 0.003, SRMR never exceeded 0.012, and the BICs of the more parsimonious model (i.e., scalar invariance) were always lower than those of the other models (i.e., metric and configural invariance; Table 4).

**Table 4:** Multigroup CFA for children’s and parents’ gender measurement invariance testing.

		CHISQ	DF	RMSEA	ΔRMSEA	SRMR	ΔSRMR	BIC
Child Gender	Configural	817.880	454.000	0.061		0.068		37613.540
	Metric	849.575	474.000	0.060	0.000	0.074	0.006	37523.640
	Scalar	892.589	494.000	0.061	0.001	0.075	0.001	37445.050
Parent gender	Configural	832.183	454.000	0.062		0.070		37505.110
	Metric	895.039	474.000	0.064	0.002	0.083	0.012	37446.370
	Scalar	977.533	494.000	0.067	0.003	0.086	0.003	37407.270

Limits: ΔRMSEA 0.015, ΔSRMR 0.030. Δs are calculated as absolute difference.

To test validity, we calculated the CPRS factor scores as means, whereas the factor scores of the CBCL and AQS were calculated as sums. Table 5 shows the correlations between the CPRS, CBCL, and AQS for the entire sample without differentiating by sex, whereas Table 6 shows the correlations separately for mothers and fathers. The correlational pattern indicated good validity of the CPRS.

**Table 5:** Correlational matrix between the CPRS, CBCL e ASQ.

	CPRS closeness		CPRS conflict		CPRS dependence		CBCL internalizing behavior		CBCL externalizing behavior		ASQ confidence		ASQ discomfort with closeness		ASQ relationships as Secondary		ASQ need for approval		ASQ preoccupation with Relationships	
Closeness	—																			
Conflict	−0.331	***	—																	
Dependence	0.011		0.302	***	—															
CBCL internalizing behavior	−0.311	***	0.412	***	0.236	***	—													
CBCL externalizing behavior	−0.327	***	0.572	***	0.201	***	0.598	***	—											
ASQ confidence	0.083		−0.178	***	−0.090		−0.141	*	−0.095		—									
ASQ discomfort with closeness	−0.146	**	0.188	***	0.207	***	0.194	**	0.142	**	−0.463	***	—							
ASQ relationships as secondary	−0.090		0.223	***	0.269	***	0.032		−0.021		−0.289	***	0.371	***	—					
ASQ need for approval	−0.131	**	0.275	***	0.209	***	0.241	***	0.117	*	−0.242	***	0.277	***	0.420	***	—			
ASQ preoccupation with relationships	−0.048		0.310	***	0.207	***	0.279	***	0.214	***	−0.250	***	0.346	***	0.262	***	0.520	***	—	

*p < 0.05, **p < 0.01, ***p < 0.001.

**Table 6:** Correlational matrix between the CPRS, CBCL e AQS separated for mothers and fathers.

	CPRS closeness	CPRS conflict	CPRS dependence	CBCL internalizing behavior	CBCL externalizing behavior	ASQ confidence	ASQ discomfort with closeness	ASQ relationships as secondary	ASQ need for approval	ASQ preoccupation with relationships
Closeness	—	**−0.29**	0.08	−0.34	**−0.26**	0.14	−0.10	0.00	−0.13	−0.05
Conflict	**−0.36**	—	**0.48**	**0.56**	**0.55**	**−0.22**	**0.26**	**0.33**	**0.28**	**0.40**
Dependence	−0.02	**0.20**	—	0.27	**0.29**	−0.09	**0.30**	**0.42**	**0.33**	**0.33**
CBCL internalizing behavior	**−0.33**	**0.40**	**0.23**	—	**0.73**	0.05	0.15	0.08	0.15	0.23
CBCL externalizing behavior	**−0.39**	**0.59**	**0.20**	**0.58**	—	−0.07	0.17	0.03	0.00	**0.21**
ASQ confidence	0.03	**−0.14**	−0.07	**−0.17**	**−0.13**	—	**−0.34**	**−0.22**	−0.12	−0.11
ASQ discomfort with closeness	**−0.17**	**0.14**	**0.16**	**0.19**	**0.15**	**−0.53**	—	**0.45**	**0.32**	**0.44**
ASQ relationships as secondary	**−0.13**	**0.16**	0.11	0.03	0.10	**−0.34**	**0.37**	—	**0.55**	**0.41**
ASQ need for approval	−0.12	**0.27**	0.11	**0.25**	**0.20**	**−0.32**	**0.25**	**0.25**	—	**0.61**
ASQ preoccupation with relationships	−0.04	**0.24**	**0.12**	**0.28**	**0.22**	**−0.36**	**0.29**	**0.16**	**0.44**	—

Values above the diagonal refer to fathers, whereas values below the diagonal refers to mother.Bold indicate *p* < 0.05.

Finally, we conducted a MANOVA with the CPRS scores for Closeness, Conflict, and Dependency as the dependent variables, and parents’ and children’s sex, as well as their interaction, as the independent variables. At the multivariate level, the effect of parental sex was significant, Pillai’s *F*(3, 431) = 3.13, *p* < 0.05, *η_p_^2^* = 0.02, whereas children’s sex, Pillai’s *F*(3, 431) = 0.39, *p* = 0.76, *η_p_^2^* = 0.00, and the interaction effect, Pillai’s *F*(3, 431) = 0.81, *p* = 0.49, *η_p_^2^* = 0.00, were not significant. At the univariate level, considering that the assumption of homoscedasticity was not respected, we used Welch’s *F*. Only significant results concerned the relationship between parents’ sex and factor Dependency, Welch’s *F*(1, 414.43) = 7.20, *p* < 0.01, Hedge’s *g* = −0.25. Mothers (*M* = 2.54, *SD* = 1.21) reported lower scores than that of fathers (*M* = 2.83, *SD* = 1.04).

## 4. Discussion

The first aim of this study was to test the factorial validity of the Italian version of the Child–Parent Relationship Scale in a cohort of Italian parents. Second, we aimed to explore the measurement invariance of the scale regarding parents’ and children’s sex. Finally, we investigated the scale’s reliability and concurrent and convergent validity by examining the associations of the CPRS-I with parents’ attachment styles and daughters’/sons’ behavioral problems.

Referring to the factorial structure of the CPRS-I, explorative and confirmative factor analyses confirmed the original three-factor structure: Closeness, Conflict, and Dependency. The CPRS-I comprised 23 items; we excluded seven items, five of which were part of the original closeness factor. In the CPRS-I, four of the five items that constitute the closeness scale refer to the partners’ feelings (the last one regards the sharing of information by the child); the sense of closeness in our sample appears to be related to the emotional sharing in the parent–child relationship, reflecting the particularly intense emotional bonds typical of the Mediterranean “strong-families” (Giannotta et al., 2013). The excluded items of the scale have some characteristics that differ from the included items: the excluded items described the topic of the question in a more general way and required the parent to infer the children’s internal states more than the included items (i.e., “My child values his/her relationship with me”; “My child tries to please me”). It seems that the Italian respondents focused their attention on items that specifically describe children’s behaviors (i.e., “My child openly shares his/her feelings and experiences with me”) or their own experiences (i.e., “My interactions with my child make me feel effective and confident as a parent”) that are easier to understand with respect to the excluded items. Moreover, our sample is constituted by working parents with a high level of education: it is possible that these parents are particularly attentive to their children’s needs and are therefore highly able both to observe them and to reflect on their own emotions in the parental relationship.

The specificity of Mediterranean cultures is also evident in the dependency scale. Mediterranean countries can be defined as “family-oriented” countries, and the relations between parent and children are characterized by warmth, friendliness, and heightened care, with mothers showing a higher level of preoccupation compared to the Eastern cultures mothers (López-de-la-Nieta et al., 2021); moreover, in Italy, children are held closer and live with their parents for a long time (Jurado Guerrero and Naldini, 1996). Our results showed that in Italian families, dependency on parents can be considered a characteristic of the parent–child relationship, a characteristic that emerges as a factor in the CPRS-I. Moreover, this result aligned with the Verschueren and Koomen (2012, 2021) model, according to which dependence is a relational construct that plays different roles in different cultures. In the CPRS-I, we excluded one item that was originally part of the Dependence Scale: “I often think about my child when at work.” In the Italian version, all other items of this scale referred to the child, whereas this is referred to the parent itself, underlying that Italian respondents evaluate the dependency level by observing children’s behaviors and not one’s thoughts.

Related to our second aim, we showed that the CPRS-I is invariant for the sex of both parents and children, indicating that the items assess the same factors for both mothers and fathers in relation to daughters and sons. Therefore, any differences between mothers and fathers can be attributed to actual variations in the responses to some items and not to the differential functioning of the scale; the same can be said for any differences between daughters and sons. Despite the differences between the attachment behavior and characteristics of mothers and fathers (Grossmann et al., 2002), the dimensions through which both parents evaluate the quality of their parental relationships are the same, indicating that in Italian culture, the sense of closeness, conflict, and dependency perceived by caregivers are important cues of the quality of the relationship for both mothers and fathers.

Regarding the third aim, we found interesting correlations between the CPRS-I and parents’ attachment styles in the dimensions assessed using the ASQ.

The ASQ results showed that “Discomfort with Closeness” and the “Need for Approval”—both dimensions of insecure attachment styles—negatively correlated with the closeness perceived in the parent–child relationship (CPRS-I); as assumed, avoidant and anxious parents experienced a low level of closeness and warmth in the relationship with their children. Correlations differentiated for sex highlighted that two insecure attachment styles, in the “Discomfort with Closeness” and “Relationship as secondary” dimensions, negatively correlated with the closeness perceived by the fathers, whereas no correlations were found regarding the mothers. If the perception of closeness involves warmth, affection, and open communication of emotions, it is possible that these characteristics (particularly warmth and affection) are independently perceived by the mothers from their attachment style because of their pivotal role in caregiving (Mancinelli et al., 2021), whereas the father’s level of involvement in child-rearing and education perception is linked to his attachment style. If Italian fathers are usually less involved in childcare than the mothers (Riem et al., 2021), and avoidant and dismissive fathers may be less involved in caregiving; thus, they perceived low levels of closeness in the relationship.

All four dimensions of insecure attachment styles assessed by the ASQ positively correlated with the conflict factor of the CPRS-I, whereas confidence (which indicates a secure style) negatively correlated with the conflict perceived by the parent. In the parent–child relationship, conflict is usually present (Maccoby, 1992) because it is part of the educational role of the adult; it is possible that confident/secure parents attribute a positive meaning to the conflict, recognize the conflict as a natural part of the relationship, and are able to manage the conflict when it appears, resulting in a low perception of the interpersonal conflict. At the same time, we can assume that parents with attachment styles characterized by avoidance and anxiety are less able to cope with conflict because of their tendency to avoid intense emotional situations or because of the high level of anxiety that the conflict elicits, so they perceive the conflict as particularly intense. Future research could combine the observation of the behavior of the two partners with the CPRS-I to verify whether it is not only the perception of conflict but also the presence of conflict itself that is different.

Finally, all four dimensions of insecure attachment styles assessed by the ASQ were positively correlated with the CPRS-I dependence factor. Dependency is defined as a developmentally inappropriate degree of overreliance and possessiveness of the child in the relationship (Koomen et al., 2012), indicating a lack of security and, consequently, difficulty in exploration. As in the case of conflict, a certain level of dependency is naturally necessary for the parent–child relationship, and parents should recognize this aspect to properly take care of the child. High levels of dependency perceived in the CPRS-I could indicate difficulty for the parent in assuming her/his caregiver role, showing anxiety about the relationship, underestimating or avoiding one’s own role, which occurs in insecure attachment styles.

The correlations between the CPRS-I and the evaluation of behavioral and emotional problems in children confirmed the results found by Driscoll and Pianta (2011)—closeness perceived in the relationship negatively correlated with both internalizing and externalizing behaviors, and conflict positively correlated with these two types of behaviors. A high sense of closeness, typical of secure attachment, seemed to be a protective factor for behavioral problems (Pianta, 1999), in which children learn to express and explore their own emotions, and parents are supportive of this process; this dynamic is related to children’s high levels of adaptive and social behavior (David and DiGiuseppe, 2016). In contrast, in relationships characterized by high levels of conflict, the expression and regulation of emotions can be less supported by adults so children tend to show more behavioral and emotional difficulties. As reported by Acar et al. (2019), conflict relationships negatively impact children’s behavior and are positively associated with their externalizing behaviors. Moreover, using the CPRS, these authors showed that parent–child closeness and conflict moderate the associations between authoritarian parenting and children’s externalizing and internalizing behaviors, respectively, confirming the important role of the parent–child relationship in behavioral problems. Finally, in our sample, dependency positively correlated with internalizing and externalizing behavioral problems—high levels of dependence seemed to indicate an anxious relationship in which the child is incapable of exploring the world and being autonomous from the parent, thus showing inappropriate behaviors.

The comparison between mothers and fathers showed that mothers consider their daughters and sons to be less dependent on them than fathers perceive them to be. Although the literature has not found significant differences between mothers and fathers in caregiving representations (Psouni, 2019), it is possible that fathers and mothers interpret children’s behaviors with different degrees of dependency. In Italy, mothers are often the main caregivers who care for their children’s daily needs, with fathers participating to a lesser extent. It is possible that mothers and fathers react to the same child’s behavior differently, with mothers considering it as part of their daily routine and fathers evaluating it as a lack of autonomy.

### 4.1. Limitations and future perspectives

This study has some limitations. The first pertains to the type of task used. In fact, a self-report scale evaluating parents’ perceptions of the relationship with the child cannot highlight the relational behaviors that are enacted. Moreover, children’s behavioral problems were assessed by the same parent who completes the CPRS-I, and in the future, it would be interesting to add a direct observation of the relationship (see Driscoll and Pianta, 2011) and of children’s behavior to verify the accuracy of subjective perception with respect to what is happening between parents and children. Moreover, the sample was not balanced in terms of sex, as most respondents (62%) were mothers (as often happens when questionnaires regard parenting). Another limitation concerns snowball sampling, which started from universities and involved mostly two parent households. In light of the important role of culture and family characteristics on the parent–child relationship, these aspects could reduce the generalizability of the results. In the future, studies may look into applying the CPRS-I with a more balanced and simple approach, considering parents’ sex, educational level, and family characteristics. A further limitation of this study is the lack of the exploration of possible differences in the parent’s perception of the relationship based on the children age. In line with previous CPRS validation studies, we used the children’s age as an inclusion criterion, but in the future, exploring differences in the parents’ perception of the relationship with respect to this data, would better delineate the quality of the parent–child relationship at different stages of children’s development. Finally, this validation investigated the parent’s perception of the relationship at a single time. In the future, scholars could carry out a longitudinal study, as done by Driscoll and Pianta (2011), for the short form of the CPRS, in order to confirm the stability of the scale in the time.

Despite the above-mentioned limitations, the CPRS-I may have relevant applications both in research and in the clinical field. The scale is simple to administer, composed of 23 items assessing the characteristics of a specific attachment relationship. In this research area, this scale may be used to assess affective relationships, complementing the attachment profiles that emerge using other tools focused on the internal working model. Therefore, a professional will be able to investigate not only the representation of attachment *per se*, but also its specific activation in the case of relationships with children, providing fundamental information to improve family relationships. In the clinical field, CPRS-I may help the therapist and patient individuate critical aspects of the affective bonds with the children and hypothesize effective modes of intervention for that specific situation. Knowing adults’ perceptions of their relationship with their children allows us to highlight the motivations that direct the relational and educational behaviors of parents themselves, providing important knowledge that can be used to improve the relationship itself. In addition, the scale can be offered at different time points in the parent–child relationship, allowing the monitoring of changes over time. This could be particularly useful in the case of children with neurodevelopmental disabilities, where parents’ perceptions of the child and the relationship may change quickly (for example, before and after a diagnosis), specifically about closeness, conflict, and dependence. Finally, the CPRS-I can be used as a specific tool in university training programs for future professionals, especially psychologists interested in the field of education.

## 5. Conclusion

In this research, we confirmed the three-factor structure of the Italian long form of the Child–Parent Relationship Scale and showed the measurement invariance of the CPRS-I regarding the sex of parents and daughters or sons. This work has contributed to individuating a validated research task that can measure the main characteristics of a specific parent–child relationship in different cultures, as Escalante-Barrios et al. (2020) pointed out, and has offered a scale that considers three core aspects of the relationship: closeness, conflict, and dependency. Moreover, this work confirmed the relationship between parents’ perceptions of their relationship with their own daughters and sons and other psychological variables, such as parents’ attachment style and children’s behavior, highlighting the importance of the quality of the parent–child relationship for both partners involved.

## Data availability statement

The raw data supporting the conclusions of this article will be made available by the authors, without undue reservation.

## Ethics statement

The studies involving humans were approved by Commissione Etica per la Ricerca in Psicologia CERPS, Università Cattolica del Sacro Cuore, Milano. The studies were conducted in accordance with the local legislation and institutional requirements. The participants provided their written informed consent to participate in this study.

## Author contributions

AV, IC, AG, and RP contributed to conception and design of the study. TR collected the data and organized the database. AG and NP performed the statistical analysis. IC, RP, and AM supervised the research. AV wrote the first draft of the manuscript. TR and NP wrote sections of the manuscript. IC, AM, and RP contributed to manuscript revision. All authors contributed to the article and approved the submitted version.

## Conflict of interest

The authors declare that the research was conducted in the absence of any commercial or financial relationships that could be construed as a potential conflict of interest.

## Publisher’s note

All claims expressed in this article are solely those of the authors and do not necessarily represent those of their affiliated organizations, or those of the publisher, the editors and the reviewers. Any product that may be evaluated in this article, or claim that may be made by its manufacturer, is not guaranteed or endorsed by the publisher.

## Footnotes

^1^Available at https://education.virginia.edu/research-initiatives/research-centers-labs/center-advanced-study-teaching-and-learning/castl-measures..
